# Class (I) Phosphoinositide 3-Kinases in the Tumor Microenvironment

**DOI:** 10.3390/cancers9030024

**Published:** 2017-03-04

**Authors:** David Gyori, Tamara Chessa, Phillip T. Hawkins, Len R. Stephens

**Affiliations:** The Babraham Institute, Cambridge CB22 3AT, UK; david.gyori@babraham.ac.uk (D.G.); tamara.chessa@babraham.ac.uk (T.C.); len.stephens@babraham.ac.uk (L.R.S.)

**Keywords:** PI3K, tumor microenvironment, solid cancer, cell signaling, PTEN

## Abstract

Phosphoinositide 3-kinases (PI3Ks) are a diverse family of enzymes which regulate various critical biological processes, such as cell proliferation and survival. Class (I) PI3Ks (PI3Kα, PI3Kβ, PI3Kγ and PI3Kδ) mediate the phosphorylation of the inositol ring at position D3 leading to the generation of PtdIns(3,4,5)P3. PtdIns(3,4,5)P3 can be dephosphorylated by several phosphatases, of which the best known is the 3-phosphatase PTEN (phosphatase and tensin homolog). The Class (I) PI3K pathway is frequently disrupted in human cancers where mutations are associated with increased PI3K-activity or loss of PTEN functionality within the tumor cells. However, the role of PI3Ks in the tumor stroma is less well understood. Recent evidence suggests that the white blood cell-selective PI3Kγ and PI3Kδ isoforms have an important role in regulating the immune-suppressive, tumor-associated myeloid cell and regulatory T cell subsets, respectively, and as a consequence are also critical for solid tumor growth. Moreover, PI3Kα is implicated in the direct regulation of tumor angiogenesis, and dysregulation of the PI3K pathway in stromal fibroblasts can also contribute to cancer progression. Therefore, pharmacological inhibition of the Class (I) PI3K family in the tumor microenvironment can be a highly attractive anti-cancer strategy and isoform-selective PI3K inhibitors may act as potent cancer immunotherapeutic and anti-angiogenic agents.

## 1. Introduction

Phosphoinositide 3-kinases (PI3Ks) phosphorylate the 3-hydroxyl group of the inositol ring leading to the generation of PtdIns(3)P, PtdIns(3,4)P2 and PtdIns(3,4,5)P3 [1]. These lipid messengers have different spatio-temporal distributions within the cell and are involved in many biological functions including survival, proliferation, metabolism, cytoskeletal rearrangement, migration and vesicular trafficking [2]. In mammals, PI3Ks are subgrouped into three unique classes based on structural and enzyme-kinetic differences [3]. The best known PI3Ks belong to the Class (I) PI3-kinase family and are termed as PI3Kα, PI3Kβ, PI3Kγ or PI3Kδ [4]. PI3Kα and PI3Kβ are ubiquitously expressed, while the PI3Kγ and PI3Kδ isoforms are enriched in hematopoietic cells, such as leukocytes [5]. The main phosphoinositide product generated by the Class (I) PI3Ks under physiological conditions is PtdIns(3,4,5)P3. PtdIns(3,4,5)P3 is a second messenger, which can activate a number of downstream molecules in the PI3K signaling pathway, including the 3-phosphoinositide dependent protein kinase-1 (PDK1), the Ser/Thr kinase AKT and the mammalian target of rapamycin complex 1 (mTORC1) [4,6]. PtdIns(3,4,5)P3 can be dephosphorylated by phosphoinositide phosphatases, such as the 3-phosphatase PTEN (phosphatase and tensin homolog) or the 5-phosphatase SHIP1 (SH2 domain-containing inositol phosphatase 1 or INPP5D).

Class (I) PI3Ks are frequently activated in human cancers where mutations are linked with cellular transformation and tumor progression. Solid cancers often exhibit elevated PI3Kα activity [7]. Abnormal activation and amplification of the PIK3CA oncogene—encoding the catalytic subunit of PI3Kα—is one of the most commonly observed events associated with malignant transformation and found to be present in multiple tumor types including breast, colon, and ovarian cancer [8]. The most frequent alterations in PI3Kα occur at specific hotspots in the coding sequence, namely the H1047R catalytic domain and the E545K and E542K helical domain mutations [9]. Oncogenic mutations have commonly been found in PI3Kα, but rarely in PI3Kγ and PI3Kδ. In the last few years, activating mutations in the gene encoding the catalytic subunit of PI3Kβ, PIK3CB, have also been described and PI3Kβ signaling has been implicated in tumorigenesis (e.g., prostate and breast cancer) [10,11]. Moreover, the catalytic activity of PI3Kβ has been shown to sustain the proliferation of PTEN-deficient cancer cells in certain tumors [12,13]. However, while PI3K signaling is often hyperactivated in solid cancers, the clinically tested PI3K inhibitors in monotherapy have shown only limited effect on tumor cells [14]. This may be due to intrinsic and acquired cancer cell resistance to PI3K inhibition, as well as the fact that tumor cells can activate parallel signaling pathways controlling growth and survival [15]. Additionally, pan–Class (I) PI3K inhibitors can cause serious adverse effects, such as hyperglycaemia and/or hyperinsulinemia in patients due to the central role of PI3Kα in glucose homeostasis, limiting the maximal effective doses that can be tolerated [16]. Exploring the role of individual PI3K isoforms in different cells of the tumor microenvironment may contribute to the design of more effective combination therapies, because these inhibitors can be tolerated at doses leading to greater effective inhibition of their targets. Further, the existence of natural isoform-selective PI3K inhibitors [17] as well as the development of new isoform-selective agents by the pharmaceutical industry [7] raise the possibility of using PI3K inhibitors as novel cancer therapeutics.

The role of PI3Ks in the tumor microenvironment however is less well understood. Solid cancers (including those of epithelial origin) consist of two distinct compartments: the tumor parenchyma—containing the neoplastic cells—and the surrounding stroma. The stroma includes fibroblasts, connective tissue, blood vessels and immune cells, all of which are mainly produced by the host and are critical for tumor growth and progression [18]. This review will focus on how dysregulation of the PI3K signaling pathway in the tumor microenvironment (including immune cells, blood vessels and fibroblasts) impacts on cancer cell growth and progression of solid tumors.

## 2. Role of PI3K in Immune Cells of the Tumor Microenvironment

Solid cancers are highly complex pathologic structures composed of the neoplastic cells and a tumor-associated microenvironment [19]. While PI3Kγ and PI3Kδ are present at low levels in many cells and tissues, they are very highly expressed in leukocytes. Under physiological conditions, PI3Kγ is responsible for many critical leukocyte responses to G protein-coupled-receptors (GPCRs), perhaps most clearly the chemotaxis and production of reactive oxygen species by neutrophils [4], while PI3Kδ is required for several leukocyte responses to tyrosine kinase-coupled receptors, for example the antigen receptors and their co-regulatory molecules which control the function and differentiation of T and B lymphocytes [5,20]. Surprisingly, given their apparent importance for an effective innate and adaptive immune response to pathogens, recent preclinical animal studies suggest that pharmacological inhibition/genetic ablation of PI3Kγ and PI3Kδ isoforms in the host can actually suppress tumor growth in a wide range of solid cancers and is not only limited to hematological malignancies [21,22,23]. Current evidence indicates that these effects are probably mediated by dominant roles for PI3Kγ and PI3Kδ in the leukocyte signaling pathways which allow tumors to suppress immune system attack. Further, considering the fact that the expression of PI3Kγ and PI3Kδ is mainly restricted to hematopoietic cells, inhibitors specifically targeting these isoforms can avoid metabolic side effects due to inhibition of PI3Kα.

### 2.1. The Role of PI3Kγ in Tumor-Associated Myeloid Cells

The sole Class IB isoform, PI3Kγ, is highly expressed in immune cells of myeloid origin, such as neutrophils and macrophages, but not in the cancer cells themselves of most solid tumors. Tumor-associated myeloid cells (TAMCs)—including tumor-associated macrophages (TAMs), tumor-associated neutrophils (TANs) and myeloid-derived suppressor cells (MDSCs)—are major cell types found in the tumor microenvironment clinically and in a wide range of preclinical tumor models [24,25]. Tumor masses can contain as many CD11b^+^ TAMCs as cancer cells and those myeloid cells can secrete anti-inflammatory cytokines which suppress immune responses [24]. PI3Kγ-deficient mice showed significantly suppressed tumor growth and metastasis formation, as well as increased host survival in a range of solid tumor models [21,26]. Moreover, pharmacological inhibition of PI3Kγ decreased cancer progression and promoted anti-tumor T-cell immune responses [22,27,28]. The activation of PI3Kγ was demonstrated to be necessary for the induction of an immunosuppressive transcriptional program in TAMCs. Inhibition of PI3Kγ reprogrammed those myeloid cells from an immunosuppressive to an immunostimulatory phenotype. This restored the numbers of functional CD8^+^ T cells in the tumor, as well as synergized with checkpoint inhibitor therapies (anti-CTLA4 and anti-PD-1 antibodies; treatments which directly interfere with additional, direct pathways by which cancer cells “switch off” CD8^+^ T cells) to promote tumor regression in syngeneic mouse models [22]. These studies suggest that targeting the PI3Kγ-dependent signaling pathways in tumor-associated myeloid cells may provide novel approaches to increase the long-term survival of cancer patients [29]. Further, the importance of PI3Kγ in the regulation of migration of neutrophil granulocytes [30], together with the identification of the pro-tumorigenic function of neutrophils [31,32], suggests PI3Kγ may play a role in TANs as well.

### 2.2. The Role of PI3Kδ in Regulatory T Cells

PI3Kδ is abundant in both lymphocytes and myeloid cells and is activated by antigen, cytokine and growth factor receptors [33]. Recent evidence has shown that genetic inactivation of PI3Kδ in mice protects against hematological tumors and also a wide range of solid cancers [23]. In addition, pharmacological inhibition of PI3Kδ significantly increased survival rates and decreased metastasis formation in different solid tumor models [23]. This immunomodulatory effect was due to the inactivation of PI3Kδ in the suppressive regulatory T cell subset, unleashing CD8^+^ cytotoxic T cells which could then induce tumor regression [23]. These findings suggest that PI3Kδ inhibitors are not only capable of blocking cancers of hematological origin but can also increase immune responses against solid tumors. Despite having remarkable effects in certain solid cancers, the success of immune checkpoint blockade therapies (anti-PD-1, anti-CTLA4 antibodies) in other tumors has been limited by the development of additional immune resistance mechanisms, for example a block in the infiltration and development of functional CD8^+^ T cells at the tumor site itself. Among these additional mechanisms, myeloid cells and regulatory T lymphocytes are thought to play a major role in limiting effective anti-tumor immunity. PI3Kγ/δ inhibitors may help overcome these problems by inhibiting the immune suppressive leukocyte subsets, such as tumor-associated macrophages and regulatory T cells.

### 2.3. The Role of PI3Ks in Other Immune Cells

Cancer cells can secrete soluble factors, which are able to shape the tumor microenvironment [32]. Macrophage differentiation is mainly driven by colony-stimulating factor-1 (CSF-1 or M-CSF) [34], and in the CSF-1-null mice macrophages are nearly completely depleted in the peripheral tissues, including the monocyte precursor-derived bone-resorbing osteoclasts (osteopetrotic, op/op mice) [35,36]. Therefore, inhibiting CSF-1 signaling is in the focus of current macrophage-targeted therapies [37]. It has been shown recently that combining PI3K inhibition with CSF-1 blockade significantly prolongs survival in animal models of glioblastoma multiforme [38].

Immune cells are common components of the tumor microenvironment [39]. However, those cells can exert both pro- and anti-tumor immune responses. Similar to regulatory T cells, dendritic cells (DCs) are able to secrete IL-10 and TGF-β to attenuate immune responses, which can be reversed by PI3Kγ inhibitor in preclinical mouse models of colon adenocarcinoma [26]. Moreover, the PI3Kδ isoform might play a role in other TAMC subsets such as TAMs and MDSCs too. On the other hand, Class (I) PI3K isoforms can be involved in anti-tumor immune responses as well, depending on the cellular context. For example, inactivation of PI3Kδ prevents the degranulation of NK cells, impairing their role in immune surveillance [40]. Similarly, degranulation defects have been described in CD8^+^ T cells derived from colon adenocarcinoma of PI3Kδ-deficient mice, dampening their cytotoxic activity [41]. The loss of PI3Kδ activity may also cause a defect in the activation and antigen-induced clonal expansion of CD8^+^ T cells [23,41]. PI3Kγ is a critical regulator of chemotaxis in innate immune cells too and therefore crucial for the elimination of pathogens [42]. Hence, the cell-specific functions of PI3Ks should be carefully considered when selecting the appropriate anti-cancer immunotherapy [43].

## 3. Role of PI3K in Angiogenesis in the Tumor Microenvironment

The ability of solid tumors to grow and progress essentially depends on new blood vessel formation. Stroma-cancer cell interactions play a crucial role in tumor neovascularization [44]. Class (I) PI3Ks are activated to some extent in nearly all cellular components of the peritumoral environment. However, recent findings have indicated that the PI3K signaling pathway is particularly important in the pathogenesis of tumor angiogenesis [44]. PI3K signaling can regulate solid tumor neovascularization either directly (through the endothelial cells) or indirectly (by cancer cells and via TAMCs). PI3Kα was documented to be the most important Class (I) PI3K isoform involved in the regulation of endothelial cells [45].

### 3.1. The Direct Role of PI3Kα in Angiogenesis

Endothelial cell proliferation, survival and maturation can be triggered by many stimuli, including vascular endothelial growth factor (VEGF) binding to the VEGF receptor (VEGFR) and angiopoietin (ANG) binding to TIE receptors. Although endothelial cells express all Class (I) PI3K isoforms, only PI3Kα is essential for vessel sprouting (45). PI3Kα is activated in the signaling pathway downstream of tyrosine kinase receptors (e.g., VEGFR) and accounts for most of the PtdIns(3,4,5)P3 generated in endothelial cells [45]. In mice with venous malformations, a PI3Kα-selective inhibitor significantly decreased pathological vessel formation by inhibiting endothelial cell proliferation [46]. PI3Kα has also been shown to be crucial for lymphatic vessel formation [47].

### 3.2. Indirect Role of PI3Ks in Angiogenesis

Besides endothelial cells, a lot of other cell types are capable of producing angiogenic factors, including cancer cells and tumor-associated myeloid cells. Class (I) PI3K isoforms play a role in these cells. In the last few years it has emerged that immune cell-mediated processes occurring at different stages of tumorigenesis are central to the development and progression of solid tumors [48]. As demonstrated earlier, the PI3Kγ isoform plays an important role in regulating the immune-suppressive TAM subset, which is a major source of VEGFα [49]. Moreover, pharmacological inhibition of PI3Kγ and PI3Kδ was described to further enhance the effect of anti-VEGF/VEGFR therapy in mouse models of pancreatic neuroendocrine and mammary tumors [50]. These findings are supported by the notion that pan-PI3K inhibitors targeting cancer, endothelial and myeloid cells have potent anti-angiogenic activity [51]. PI3Kβ has been shown to be the dominant isoform in platelets and plays a critical role in platelet activation and thrombus formation [52]. The activation of platelets and the coagulation system have an important function in cancer progression. The contribution of platelets to tumor cell survival in the blood highlights their key role in the development of metastases [53]. PI3Kβ inhibitors may possibly be able to shape the tumor microenvironment within the bloodstream by inhibiting the tumor cell-protective function of platelets and limit the establishment of new secondary lesions [53].

## 4. Role of PI3K in Stromal Fibroblasts of the Tumor Microenvironment

Fibroblasts constitute a major cellular component of the tumor microenvironment, and are important regulators of normal tissue homeostasis under physiological conditions by secreting various cytokines and growth factors. Cancer-associated fibroblasts (CAFs) can produce a number of paracrine factors which influence cell proliferation and survival via altering the composition of the extracellular matrix (ECM), and by changing the tumor microenvironment [54]. CAFs promote tumor progression, but the role of PI3Ks in the regulation of CAF-tumor cell interactions is less well understood. It was shown that an indirect action of a PI3Kγ inhibitor (through TAMs) decreased collagen production from CAFs [55] and accumulating evidence indicates that PI3Ks control the secretion of matrix metalloproteinases (MMP) by fibroblasts, which is crucial for tumor cell migration [56]. Further, inactivation of PTEN in stromal fibroblasts has been shown to promote mammary epithelial tumor development and progression [57]. ECM remodeling and metastasis are connected processes which contribute to cancer dissemination and PI3K signaling seems to be important for both. However, further studies are required to fully evaluate the role of Class (I) PI3K isoforms in cancer-associated fibroblasts.

## 5. Conclusions and Perspectives

**Figure 1:**
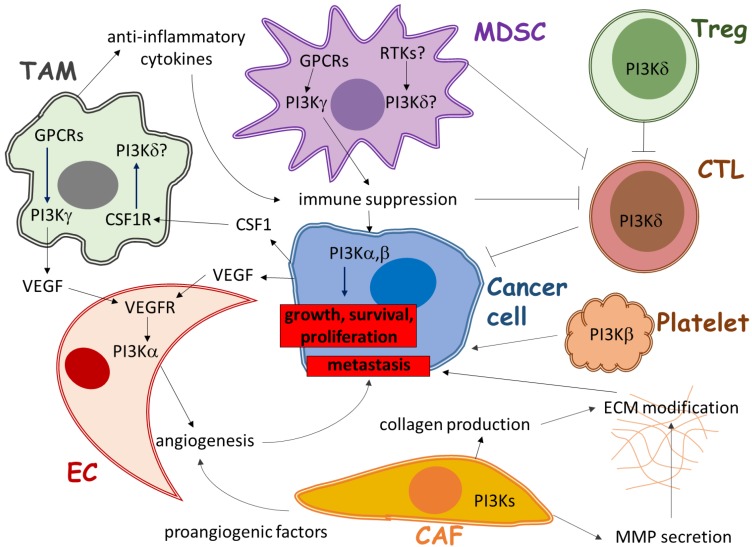
Cellular composition of the tumor microenvironment and the role of Class (I) phosphoinositide 3-kinase (PI3K) isoforms in the stromal cells which support cancer growth and progression. TAM: tumor-associated macrophage, MDSC: myeloid-derived suppressor cell, Treg: regulatory T lymphocyte, CAF: cancer-associated fibroblast, EC: endothelial cell, CTL: cytotoxic T lymphocyte, GPCR: G protein-coupled receptor, RTK: receptor tyrosine kinase.

The PI3K signaling pathway is both active in cancer cells and the tumor microenvironment and regulates not only cancer growth but also tumor protective immune responses, neovascularization and cancer-induced matrix-reorganization [58]. Class (I) PI3K isoforms are expressed in all of the different cell types in the peritumoral environment and are critical regulators of both physiological and pathophysiological cellular responses (Figure 1). However, clinical trials with PI3K inhibitors used as a monotherapy have shown only limited potential to directly arrest tumor growth, possibly as a consequence of cancer cell resistance mechanisms and drug tolerability in patients due to narrow therapeutic index [15].

In the past few years, the identification of specific and non-redundant roles for Class (I) PI3K isoforms in the tumor-protective microenvironment has raised the possibility of using isoform-selective PI3K inhibitors to downregulate the supportive stimuli derived from the stroma. This effect is exemplified by the PI3Kδ inhibitor, idelalisib, which has been approved by the FDA for the treatment of hematological malignancies. In chronic lymphocytic leukemia (CLL), PI3Kδ-inhibition interferes with the survival signals provided by stromal cells for the transformed B lymphocytes [33]. This principle may be further extended to solid tumors, where inhibition of the leukocyte-specific PI3Kγ and PI3Kδ isoforms may block immune-suppressive tumor-associated myeloid and regulatory T cells, respectively [21,22,23]. In this context, inhibition of PI3Kγ and PI3Kδ in preclinical animal models has been shown to reshape the immune response to cancer and enhance cytotoxic T lymphocyte-mediated tumor elimination, without targeting the cancer cells directly. However, the ability of PI3Kδ inhibition to modulate immune responses is probably not limited to the dysfunction of the regulatory T cell subset but may also cause a defect in CD8^+^ lymphocyte responses [23,41]. As a consequence, the level of the dependence of the tumor on key immune suppressive cells as well as the degree of the impairment of the effector T cell response must be considered together to estimate the effect of PI3Kδ inhibition on cancer growth and progression. Moreover, there are a number of documented cases where patients treated with idelalisib developed acute toxicities [59,60,61]. PI3Kδ inhibition caused several adverse effects, among which the risks of pneumonitis, diarrhea, colitis, rash, liver inflammation, neutropenia, and opportunistic infections were associated with idelalisib treatment—critically, a number of deaths among participants put new trials on hold [62]. In at least some of these cases, these adverse effects may be due to on-target effects and the development of a “hyper-active” immune response. To be able to overcome such complications, the administration of PI3K isoform-selective drugs below their maximum-tolerated dose, most likely in combination with other treatments that act on parallel pathways (e.g., “checkpoint” inhibitors or CSF-1 blockade), might help to avoid complete immune system deregulation. These strategies may also minimize the risk of the development of tumor-intrinsic resistance.

PI3K signaling also has pleiotropic roles in angiogenesis, which can provide a rationale for using PI3K inhibitors as anti-angiogenic agents [44]. Evidence from the literature suggests that PI3K inhibition has a modulatory effect on the tumor vasculature [52]. Moreover, the PI3Kα isoform has been implicated in the regulation of critical endothelial cell functions [45]. However, the underlying mechanisms are not fully understood and there is a concern that PI3Kα inhibitors will cause toxicity through interfering with insulin signaling. Additional preclinical studies are required to evaluate the potential for inhibiting different PI3K isoforms in cancer-associated fibroblasts.

In summary, Class (I) PI3K isoforms play critical, but cell-specific roles both in cancer cells and in the tumor microenvironment. As a consequence, a precision medicine based approach should be considered when designing the appropriate therapy and drug combinations where tumor-stromal cell interactions are taken into account as well [63,64]. This approach which would rely on defining the most useful biomarkers to direct the pre-clinical studies and stratify patients.

## References

[1] Hawkins P., Stephens L.R. (2015). PI3K signalling in inflammation. Biochim. Biophys. Acta.

[2] Vanhaesebroeck B., Stephens L., Hawkins P. (2012). PI3K signalling: the path to discovery and understanding. Nat. Rev. Mol. Cell Biol..

[3] Vanhaesebroeck B., Guillermet-Guibert J., Graupera M., Bilanges B. (2010). The emerging mechanisms of isoform-specific PI3K signalling. Nat. Rev. Mol. Cell Biol..

[4] Hawkins P., Anderson K.E., Davidson K., Stephens L.R. (2006). Signalling through class I PI3Ks in mammalian cells. Biochem. Soc. Trans..

[5] Okkenhaug K. (2013). Signaling by the phosphoinositide 3-kinase family in immune cells. Annu. Rev. Immunol..

[6] Cantley L.C. (2002). The phosphoinositide 3-kinase pathway. Science.

[7] Fruman D., Rommel C. (2014). PI3K and cancer: lessons, challenges and opportunities. Nat. Rev. Drug Discov..

[8] Thorpe L., Yuzugullu H., Zhao J.J. (2015). PI3K in cancer: Divergent roles of isoforms, modes of activation and therapeutic targeting. Nat. Rev. Cancer.

[9] Samuels Y., Diaz L.A., Schmidt-Kittler O., Cummins J.M., Delong L., Cheong I., Rago C., Huso D.L., Lengauer C., Kinzler K.W. (2005). Mutant PIK3CA promotes cell growth and invasion of human cancer cells. Cancer Cell.

[10] Hill K., Kalifa S., Das J.R., Bhatti T., Gay M., Williams D., Taliferro-Smith L., De Marzo A.M. (2010). The role of PI 3-kinase p110beta in AKT signally, cell survival, and proliferation in human prostate cancer cells. Prostate.

[11] Dbouk H., Khalil B.D., Wu H., Shymanets A., Nürnberg B., Backer J.M. (2013). Characterization of a tumor-associated activating mutation of the p110β PI 3-kinase. PLoS ONE.

[12] Wee S., Wiederschain D., Maira S.M., Loo A., Miller C., deBeaumont R., Stegmeier F., Yao Y.M., Lengauer C. (2008). PTEN-deficient cancers depend on PIK3CB. Proc. Natl. Acad. Sci. USA.

[13] Jiang X., Chen S., Asara J.M., Balk S.P. (2010). Phosphoinositide 3-kinase pathway activation in phosphate and tensin homolog (PTEN)-deficient prostate cancer cells is independent of receptor tyrosine kinases and mediated by the p110beta and p110delta catalytic subunits. J. Biol. Chem..

[14] Engelman J. (2009). Targeting PI3K signalling in cancer: Opportunities, challenges and limitations. Nat. Rev. Cancer.

[15] Brown K., Toker A. (2015). The phosphoinositide 3-kinase pathway and therapy resistance in cancer. F1000Prime Rep..

[16] Busaidy N., Farooki A., Dowlati A., Perentesis J.P., Dancey J.E., Doyle L.A., Brell J.M., Siu L.L. (2012). Management of metabolic effects associated with anticancer agents targeting the PI3K-Akt-mTOR pathway. J. Clin. Oncol..

[17] Safdari Y., Khalili M., Ebrahimzadeh M.A., Yazdani Y., Farajnia S. (2015). Natural inhibitors of PI3K/AKT signaling in breast cancer: emphasis on newly-discovered molecular mechanisms of action. Pharmacol. Res..

[18] Dworak H.F. (1986). Tumors: Wounds that do not heal. Similarities between tumor stroma generation and wound healing. N. Engl. J. Med..

[19] McAllister S., Weinberg R.A. (2014). The tumour-induced systemic environment as a critical regulator of cancer progression and metastasis. Nat. Cell Biol..

[20] Lucas C., Chandra A., Nejentsev S., Condliffe A.M., Okkenhaug K. (2016). PI3Kδ and primary immunodeficiencies. Nat. Rev. Immunol..

[21] Kaneda M., Messer K.S., Ralainirina N Li H., Leem C.J., Gorjestani S., Woo G., Nguyen A.V., Figueiredo C.C., Foubert P. (2016). PI3Kγ is a molecular switch that controls immune suppression. Nature.

[22] De Henau O., Rausch M., Winkler D., Campesato LF Liu C., Cymerman D.H., Budhu S., Ghosh A., Pink M., Tchaicha J. (2016). Overcoming resistance to checkpoint blockade therapy by targeting PI3Kγ in myeloid cells. Nature.

[23] Ali K., Soond D.R., Pineiro R., Hagemann T., Pearce W., Lim E.L., Bouabe H., Scudamore C.L., Hancox T., Maecker H. (2014). Inactivation of PI(3)K p110delta breaks regulatory T-cell-mediated immune tolerance to cancer. Nature.

[24] Mosely S.I., Prime J.E., Sainson R.C., Koopmann J.O., Wang D.Y., Greenawalt D.M., Ahdesmaki M.J., Leyland R., Mullins S., Pacelli L. (2017). Rational Selection of Syngeneic Preclinical Tumor Models for Immunotherapeutic Drug Discovery. Cancer Immunol. Res..

[25] Zhang Q., Liu L., Gong C.Y., Shi H.S., Zeng Y.H., Wang X.Z., Zhao Y.W., Wei Y.Q. (2012). Prognostic significance of tumor-associated macrophages in solid tumor: a meta-analysis of the literature. PLoS ONE.

[26] Gonzalez-Garcia A., Sanchez-Ruiz J., Flores J.M., Carrera A.C. (2010). Phosphatidylinositol 3-kinase gamma inhibition ameliorates inflammation and tumor growth in a model of colitis-associated cancer. Gastroenterology.

[27] Schmid M.C., Avraamides C.J., Dippold H.C., Franco I., Foubert P., Ellies L.G., Acevedo L.M., Manglicmot J.R., Song X., Wrasidlo W. (2011). Receptor tyrosine kinases and TLR/IL1Rs unexpectedly activate myeloid cell PI3kgamma, a single convergent point promoting tumor inflammation and progression. Cancer Cell.

[28] Schmid M., Franco I., Kang S.W., Hirsch E., Quilliam L.A., Varner J.A. (2013). PI3-kinase gamma promotes Rap1a-mediated activation of myeloid cell integrin alpha4beta1, leading to tumor inflammation and growth. PLoS ONE.

[29] Khalil D., Smith E.L., Brentjens R.J., Wolchok J.D. (2016). The future of cancer treatment: immunomodulation, CARs and combination immunotherapy. Nat. Rev. Clin. Oncol..

[30] Hawkins P., Stephens L.R. (2007). PI3Kgamma is a key regulator of inflammatory responses and cardiovascular homeostasis. Science.

[31] Coffelt S.B., Kersten K., Doornebal C.W., Weiden J., Vrijland K., Hau C.S., Verstegen N.J., Ciampricotti M., Hawinkels L.J., Jonkers J. (2015). IL-17-producing γδ T cells and neutrophils conspire to promote breast cancer metastasis. Nature.

[32] Spiegel A., Brooks M.W., Houshyar S., Reinhardt F., Ardolino M., Fessler E., Chen M.B., Krall J.A., DeCock J., Zervantonakis I.K. (2016). Neutrophils Suppress Intraluminal NK Cell-Mediated Tumor Cell Clearance and Enhance Extravasation of Disseminated Carcinoma Cells. Cancer Discov..

[33] Okkenhaug K., Burger J.A. (2016). PI3K signaling in normal B cells and chronic lymphocytic leukemia (CLL). Curr. Top Microbiol. Immunol..

[34] Chitu V., Stanley E.R. (2006). Colony-stimulating factor-1 in immunity and inflammation. Curr. Opin. Immunol..

[35] Yoshida H., Hayashi S., Kunisada T., Ogawa M., Nishikawa S., Okamura H., Sudo T., Shultz L.D., Nishikawa S. (1990). The murine mutation osteopetrosis is in the coding region of the macrophage colony stimulating factor gene. Nature.

[36] Wiktor-Jedrzejczak W., Bartocci A., Ferrante A.W., Ahmed-Ansari A., Sell K.W., Pollard J.W., Stanley E.R. (1990). Total absence of colony-stimulating factor 1 in the macrophage-deficient osteopetrotic (op/op) mouse. Proc. Natl. Acad. Sci. USA.

[37] Quail D., Joyce J.A. (2017). Molecular Pathways: Deciphering Mechanisms of Resistance to Macrophage-Targeted Therapies. Clin. Cancer Res..

[38] Quail D., Bowman R.L., Akkari L., Quick M.L., Schuhmacher A.J., Huse J.T., Holland E.C., Sutton J.C., Joyce J.A. (2016). The tumor microenvironment underlies acquired resistance to CSF-1R inhibition in gliomas. Science.

[39] Coussens L., Zitvogel L., Palucka A.K. (2013). Neutralizing tumor-promoting chronic inflammation: A magic bullet?. Science.

[40] Zebedin E., Simma O., Schuster C., Putz E.M., Fajmann S., Warsch W., Eckelhart E., Stoiber D., Weisz E., Schmid J.A. (2008). Leukemic challenge unmasks a requirement for PI3Kdelta in NK cell-mediated tumor surveillance. Blood.

[41] Putz E., Prchal-Murphy M., Simma O.A., Forster F., Koenig X., Stockinger H., Piekorz R.P., Freissmuth M., Müller M., Sexl V. (2012). PI3Kdelta is essential for tumor clearance mediated by cytotoxic T lymphocytes. PLoS ONE.

[42] Hirsch E., Katanaev V.L., Garlanda C., Azzolino O., Pirola L., Silengo L., Sozzani S., Mantovani A., Altruda F., Wymann M.P. (2000). Central role for G protein-coupled phosphoinositide 3-kinase gamma in inflammation. Science.

[43] Spitzer M., Carmi Y., Reticker-Flynn N.E., Kwek S.S., Madhireddy D., Martins M.M., Gherardini P.F., Prestwood T.R., Chabon J., Bendall S.C. (2017). Systemic Immunity Is Required for Effective Cancer Immunotherapy. Cell.

[44] Soler A., Angulo-Urarte A., Graupera M. (2015). PI3K at the crossroads of tumor angiogenesis signaling pathways. Mol. Cell Oncol..

[45] Graupera M., Guillermet-Guibert J., Foukas L.C., Phng L.K., Cain R.J., Salpekar A., Pearce W., Meek S., Millan J., Cutillas P.R. (2008). Angiogenesis selectively requires the p110alpha isoform of PI3K to control endothelial cell migration. Nature.

[46] Castel P., Carmona F.J., Grego-Bessa J., Berger M.F., Viale A., Anderson K.V., Bague S., Scaltriti M., Antonescu C.R., Baselga E. (2016). Somatic PIK3CA mutations as a driver of sporadic venous malformations. Sci. Transl. Med..

[47] Stanczuk L., Martinez-Corral I., Ulvmar M.H., Zhang Y., Lavina B., Fruttiger M., Adams R.H., Saur D., Betsholtz C., Ortega S. (2015). cKit lineage hemogenic endothelium-derived cells contribute to mesenteric lymphatic vessels. Cell Rep..

[48] Gajewski T., Schreiber H., Fu Y.X. (2013). Innate and adaptive immune cells in the tumor microenvironment. Nat. Immunol..

[49] Stockmann C., Doedens A., Weidemann A., Zhang N., Takeda N., Greenberg J.I., Cheresh D.A., Johnson R.S. (2008). Deletion of vascular endothelial growth factor in myeloid cells accelerates tumorigenesis. Nature.

[50] Rivera L., Meyronet D., Hervieu V., Frederick M.J., Bergsland E., Bergers G. (2015). Intratumoral myeloid cells regulate responsiveness and resistance to antiangiogenic therapy. Cell Rep..

[51] Schmid M., Varner J.A. (2010). Myeloid cells in the tumor microenvironment: Modulation of tumor angiogenesis and tumor inflammation. J. Oncol..

[52] Martin V., Guillermet-Guibert J., Chicanne G., Cabou C., Jandrot-Perrus M., Plantavid M., Vanhaesebroeck B., Payrastre B., Gratacap M.P. (2010). Deletion of the p110beta isoform of phosphoinositide 3-kinase in platelets reveals its central role in Akt activation and thrombus formation in vitro and in vivo. Blood.

[53] Gay L., Felding-Habermann B. (2011). Contribution of platelets to tumour metastasis. Nat. Rev. Cancer.

[54] Hirsch E., Ciraolo E., Franco I., Ghigo A., Martini M. (2014). PI3K in cancer-stroma interactions: bad in seed and ugly in soil. Oncogene.

[55] Kaneda M., Cappello P., Nguyen A.V., Ralainirina N., Hardamon C.R., Foubert P., Schmid M.C., Sun P., Mose E., Bouvet M. (2016). Macrophage PI3Kgamma drives pancreatic ductal adenocarcinoma progression. Cancer Discov..

[56] Awad A., Kandalam V., Chakrabarti S., Wang X., Penninger J.M., Davidge S.T., Oudit G.Y., Kassiri Z. (2009). Tumor necrosis factor induces matrix metalloproteinases in cardiomyocytes and cardiofibroblasts differentially via superoxide production in a PI3Kgamma dependent manner. Am. J. Physiol. Cell Physiol..

[57] Trimboli A., Cantemir-Stone C.Z., Li F., Wallace J.A., Merchant A., Creasap N., Thompson J.C., Caserta E., Wang H., Chong J.L. (2009). Pten in stromal fibroblasts suppresses mammary epithelial tumours. Nature.

[58] Okkenhaug K., Graupera M., Vanhaesebroeck B. (2016). Targeting PI3K in Cancer: Impact on Tumor Cells, Their Protective Stroma, Angiogenesis, and Immunotherapy. Cancer Discov..

[59] Louie C., DiMaio M.A., Matsukuma K.E., Coutre S.E., Berry G.J., Longacre T.A. (2015). Idelalisib-associated enterocolitis: Clinicopathologic features and distinction from other enterocolitides. Am. J. Surg. Pathol..

[60] Weidner A., Panarelli N.C., Geyer J.T., Bhavsar E.B., Furman R.R., Leonard J.P., Jessurun J., Yantiss R.K. (2015). Idelalisib-associated colitis: Histologic findings in 14 patients. Am. J. Surg. Pathol..

[61] Coutré S.E., Barrientos J.C., Brown J.R., de Vos S., Furman R.R., Keating M.J., Li D., O’Brien S.M., Pagel J.M., Poleski M.H. (2015). Management of adverse events associated with idelalisib treatment: Expert panel opinion. Leuk. Lymphoma.

[62] U.S. Food and Drug Administration FDA Alerts Healthcare Professionals about Clinical Trials with Zydelig (idelalisib) in Combination with other Cancer Medicines. http://www.fda.gov/Drugs/DrugSafety/ucm490618.htm.

[63] Hackl H., Charoentong P., Finotello F., Trajanoski Z. (2016). Computational genomics tools for dissecting tumour-immune cell interactions. Nat. Rev. Genet..

[64] Charoentong P., Finotello F., Angelova M., Mayer C., Efremova M., Rieder D., Hackl H., Trajanoski Z. (2017). Pan-cancer Immunogenomic Analyses Reveal Genotype-Immunophenotype Relationships and Predictors of Response to Checkpoint Blockade. Cell Rep..

